# Hepatitis B Virus X Protein-Induced ROR*γ* Expression to Promote the Migration and Proliferation of Hepatocellular Carcinoma

**DOI:** 10.1155/2019/5407126

**Published:** 2019-11-03

**Authors:** Yu Huang, Huasheng Liang, Chuanxiong He, Fang Peng

**Affiliations:** ^1^Department of Hepatobiliary Surgery, The Guangxi Zhuang Autonomous Region People's Hospital, Nanning 530021, China; ^2^Beihai Institute of Endocrine and Metabolic Diseases, Ninth Affiliated Hospital of Guangxi Medical University, Beihai 536000, China; ^3^Guangxi Beihai Second People's Hospital, Beihai 536000, China

## Abstract

Aberrant expression of ROR*γ* is implicated in cancer development. A previous study identified that ROR*γ* functions as a tumor promoter to drive hepatocellular carcinoma (HCC) growth. However, its expression and significance in HCC remain unclear. The central finding of this work is that ROR*γ* was overexpressed in HCC due to its dysfunction of promoter methylation, and hepatitis B virus X protein (HBx) can remarkably induce the expression of ROR*γ* in hepatocellular carcinoma through enhancing the transcriptional function. Also, the HBx-induced ROR*γ* could promote the migration and proliferation of hepatoma cells. Hence, these results suggest that ROR*γ* was an important regulator in HCC, and our finding provides new insights into the significance of ROR*γ* in HCC.

## 1. Introduction

The retinoic acid receptor-related orphan receptors (RORs) are members of nuclear receptor superfamily, including three subtypes (ROR*α*, ROR*β*, and ROR*γ*), which are a class of ligand-dependent transcriptional factors [1]. Previous studies have revealed that RORs have critical roles in the regulation of physiological and pathological processes, such as development, circadian rhythm, and cellular metabolism, especially in the immune response regulation [2–5]. Besides, ROR*γ*t is also a member of the ROR family, which is different from ROR*γ* in the N-terminal [6]. ROR*γ*t is overexpressed in the thymus, and ROR*γ* is normally expressed in the kidney, liver, skeletal muscles, adipose tissue, pancreas, and so on [7, 8]. The pathogenic mechanism studies have revealed that ROR*γ*t regulates the differentiation of Th17 cells, which is a kind of cells that secrete interleukin 17 (IL-17), a regulator of proinflammatory signaling [9, 10]. Summary of the previous reports confirm the role of ROR*γ*t in the regulation of immune-inflammatory effect. However, the study of ROR*γ* in the regulation of signaling pathway remains unclear.

In recent years, the study of ROR*γ* in cancer has received more attention. Muscat and colleagues demonstrated ROR*γ* expression is downregulated in breast tumor tissues ROR*γ* negatively regulates TGF*β*/EMT and MaSC pathways, and agonist targeting ROR*γ* could inhibit the migration and proliferation of breast cancer cells [11, 12]. However, its expression was reported to be highly expressed in non-small-cell lung cancer, its expression has a positive correlation with the lymph node metastasis, and the high-expression of ROR*γ* showed a poor prognosis [13], suggesting that ROR*γ* may perform a different function in different cancers. More interestingly, another study in breast cancer revealed that the high ROR*γ* expression represented a low survival rate [14]; therefore, the different functions of ROR*γ* were illuminated in breast cancer. And a similar phenomenon was reported in melanoma. Kupper's report showed the growth of melanoma cells was inhibited in the ROR*γ*-deficient mice [15]. ROR*γ* expression showed inverse correlation with melanoma progression [16]. Accordingly, the explanation of different functions in cancers may be due to the tumor microenvironment, and the study of ROR*γ* in cancers is worthy of further research.

Hepatocellular carcinoma (HCC) is a main malignant tumor in the digestive system, contributed to the fifth leading cause of cancer-related death [17]. ROR*γ*t was reported to be overexpressed in peripheral blood mononuclear cells of HCC patients [18], and the contribution of ROR*γ* in HCC still has not been reported. In this work, we firstly examined the expression of ROR*γ* in HCC and evaluated the potential mechanism. As hepatitis B virus (HBV) infection is a major risk factor for HCC and hepatitis B virus X protein (HBx) is a major protein in the occurrence and development of HCC [19–21], whether ROR*γ* is involved in the regulation of HBV-related HCC remains unclear. To formulate the hypothesis, we examined the expression of ROR*γ* in the tumor and adjacent tissues and found the hypomethylation of ROR*γ* in the liver tumor, and ROR*γ* expression was further enhanced in the HCC patients with HBV infection. The most important work is that HBx could induce ROR*γ* expression by promoting its transcriptional function. The biological function study demonstrated overexpression of ROR*γ* can enhance the migration and growth activity of liver cancer cells. Our finding provides new insights into the role of ROR*γ* in HCC.

## 2. Materials and Methods

### 2.1. Clinical Liver Cancer Tissues and Cell Lines

The liver cancer tissues and corresponding adjacent tissues were collected in the department of hepatobiliary surgery, the Guangxi Zhuang Autonomous Region Peopleʼs Hospital, from the patients who have not undergone any treatment, including chemotherapy and radiotherapy. The tissues were used for the analysis of the ROR*γ* expression. The study was approved by the institutional research ethics committee at the Guangxi Zhuang Autonomous Region People's Hospital (GuangXi Science and Technology-2018-30). All patients signed the written consent.

### 2.2. Cell Culture

The HepG2, SMMC-7721, HEK293T, and Huh7 cell lines were obtained from ATCC (Rockville, MD, USA). HCCLM3 cells were purchased from Shanghai Cell Bank of the Chinese Academy of Sciences (Shanghai, China). These cell lines were cultured in Dulbecco's modified Eagle medium (DMEM) (GIBCO, USA), supplemented with 10% fetal bovine serum (FBS, GIBCO, USA) with heat inactivation and 100 U/mL penicillin (GIBCO, USA). The cells were cultured in a humidified incubator with 37°C and 5% CO_2_. The passenger of cell lines was no more than 10 times, and liquid nitrogen was used for the storage of cells.

### 2.3. Western Blotting

The clinical tissues were homogenized with tissue homogenization treatment and centrifuged at 12000 g/min at 4°C for purification of protein. The cells were lysed with RIPA lysis buffer containing the protease inhibitor cocktail (Yeasen, Shanghai, China) on ice for 30 min. The lysis was centrifuged at 4°C for 15 min, and the supernatant was collected and subjected to BCA assay for protein concentration evaluation. A total of 30 *μ*g protein was subjected to SDS-PAGE and transferred to the PVDF membrane. The membrane was incubated with primary antibody overnight and subjected to the second antibody at room temperature for 1 h. The antibodies are as follows: anti-ROR*γ* (Abcam, Cambridge, USA, ab78007), anti-GAPDH (Proteintech, USA, 60004-1-Ig), anti-Myc (Santa Cruz, CA, USA, 9E10), anti-HBx (Abcam, Cambridge, USA, ab203540), and anti-*β*-actin (Proteintech, USA, 23660-1-AP). The enhanced chemiluminescence (ECL) system (Yeasen, Shanghai, China) was used to visualize the protein band. The Quantity One software (Bio-Rad, CA, USA) was used to quantify protein expression.

### 2.4. Bioinformatics Analysis of TCGA Database

The Cancer Genome Atlas (TCGA) program for liver cancer [22] was assessed in this study. A total of 59 normal liver tissues and 97 liver tumor tissues were compared in its mRNA levels. The relative expression levels were evaluated by the median-centered intensity method. The methylation analysis was analyzed with 450k methylation array, and the ratio of the methylation levels between liver tumor and corresponding adjacent normal tissues was calculated. And, the median value of methylation level was subjected to those normal liver tissues due to deficiency. Also, the correlation between the methylation level and gene mRNA expression was examined in liver tumor tissues and adjacent normal tissues.

### 2.5. *Oncomine* Analysis

The Guichard Liver microarray data were selected from the *Oncomine* portal [23]. A total of 86 normal liver tissues and 99 liver tumor tissues were analyzed by a reporter (01-150058071), and the data type was mRNA level. Furthermore, 81 cases of liver cancer patients with hepatitis virus infection information were classified into two groups, 48 patients without HBV infection and 33 patients with HBV infection.

### 2.6. Quantitative Real-Time Reverse Transcription PCR (qRT-PCR)

The cells were washed with cold phosphate buffer solution (PBS) twice and collected with a cell scraper. The cells were lysed with TRIzol RNA extraction reagent (Invitrogen, Gaithersburg, MD) for 10 min at room temperature, and trichloromethane was subsequently added. After vortex oscillation, the reaction solution was stored at 4°C for 10 min and centrifuged at 4°C for 10 min. The supernatant was collected and mixed with isopropanol to precipitate total RNA. With 10 min incubation, the solution was centrifuged for 10 min, and the collected RNA was washed with 75% ethanol. The first-strand cDNA was synthesized with SuperScript reverse transcriptase (Invitrogen, MD, USA). The SYBR green-quantitative polymerase chain reaction (PCR) system (TaKaRa, Shiga, Japan) was used to examine the mRNA levels of indicated genes in the StepOne Real-time PCR system (Applied Biosystems, USA). The relative gene expression was calculated by 2^−ΔΔCT^. The primer sequences are as follows: ROR*γ* forward, 5′-GTG GGG ACA AGT CGT CTG G-3′, ROR*γ* reverse, 5′-AGT GCT GGC ATC GGT TTC G-3′, the amplicon size is 156 bp. GAPDH forward, 5′-AGG TCG GAG TCA ACG GAT TT-3′, GAPDH reverse, 5′-ATC TCG CTC CTG GAA GAT GG-3'. GAPDH is a housekeeping gene to normalize the relative gene expression.

### 2.7. Luciferase Reporter Assay

The luciferase reporter system of ROR*γ* was conducted as follows. The promoter sequence of ROR*γ* was obtained for GeneCopoeia (Rockville, MD, USA), a web portal for promoter region clones (http://www.genecopoeia.com) [24]. The detailed ROR*γ* promoter sequence in this study contains the core promoter sequence of ROR*γ*, which was reported previously [8]. The following primers were used for amplification: forward primer (upstream position-1492), 5′-CCC TCG AGC TCC CCT GCA CTC CCA CGC C-3′, reverse primer (downstream position +93), and 5′- CCC AAG CTT GGT GCC GTC CTG GCT GCC C-3'. The sequence was inserted into a pGL3-basic plasmid (Promega, USA) with XhoI and HindIII restriction site. The genomic DNA of HepG2 cells was used as a template to amplify the promoter sequence of ROR*γ*. The HepG2 and Huh7 cells were transfected with HBx-overexpressing plasmid or siRNA against HBx (Sigma, Saint Louis, USA), combined with the luciferase reporter plasmid of ROR*γ* and Rellina plasmid. After transfection for 24 h, the cells were collected and washed with cold PBS twice. The dual-luciferase reporter system (Promega, USA) was used to examine the luciferase activity. In detail, the collected cells were lysed with a reporter lysis buffer for 30 min on ice. After lysis, the supernatant was transferred into white 96-well microplates, and the firefly luciferase solution was added, followed by the addition of Rellina luciferase substrate. The microplate was then placed into a microplate reader to examine the luciferase reporter activity.

### 2.8. Construction of Stable Overexpressing Cell with ROR*γ* and HBx

The full sequence of ROR*γ* and HBx was cloned into a pCDH-CMV-MCS-EF1-Puro vector. The cDNA of HepG2 cells was used as a template for ROR*γ* amplification, and the cDNA of HBx was cloned from a pGFP-HBx plasmid (Addgene, Cat.No.65463). The primer of ROR*γ* is as follows: forward, 5′- GCT CTA GAA TGG ACA GGG CCC CAC AGA G-3′, reverse, 5′- CGG AAT TCT CAC TTG GAC AGC CCC ACA GG-3′, and the restriction sites were XbaI and EcoRI. Then, the lentivirus plasmid of ROR*γ* or HBx combined with the packaging plasmid pCMV-DR8.91 and pCMV-VSV-G was transfected into HEK293T cells. For further incubation with 48 h, the lentiviral particles were collected, and the particles were condensed with lentivirus concentration solution. Also, the quality of particles was evaluated with the viral titer, and the qualified lentiviral particles were stored in an ultra-low temperature freezer. Then, the viral particle containing ROR*γ* and HBx was applied to incubate the indicated cells, supplemented with polybrene at the concentration of 5 *μ*g/mL. The puromycin selection (Thermo Scientific, Madison, WI, USA) was conducted to screen the positive cells.

### 2.9. Wound-Healing Assay

The cells were plated in 6-well plates and cultured for 24 h and then subjected to the lentiviral transduction particles packing ROR*γ* overexpression and control vector system for 12 h transfection. The scratches were produced across the cell monolayers with yellow tips. And the cells were washed with PBS solution for three times to remove the shedding cells. Then, the cells were cultured in fresh culture medium for another 48 h, and the confluence of cells was recorded with PrimoStar microscope (Zeiss, Jena, Germany). The migration index was calculated with the ratio of the scratch area between ROR*γ* overexpression and vector cells by Image J software (US National Institutes of Health, Bethesda, MD, USA).

### 2.10. CCK-8 Cell Viability Assay

The HepG2 cells were seeded in 96-well plates at a density of 5000 cells per well, and the cells were cultured in the indicated culture medium, supplemented with lentiviral particles of ROR*γ* overexpression and control vector. The cells were cultured for a different time, and before 3 h of indicated detection time, 10 *μ*L CCK-8 reagent (Yeasen, Shanghai, China) was added into the cells, and the absorbance value was detected by a microplate reader (Thermo Scientific, Madison, WI, USA) at 450 nm.

### 2.11. Colony Formation Assay

HepG2 cells overexpressing ROR*γ* and with vector control cells were seeded in a 3.5 cm dish (500 cells/dish) and cultured for 2 weeks in DMEM (containing 10% FBS). After washing twice gently with PBS, cells fixed with 4% paraformaldehyde and stained with crystal violet. The number of foci containing ≥50 cells was calculated at 40X magnification using an optical microscope (Zeiss, Jena, Germany).

### 2.12. Statistical Analysis

Data are represented as mean ± standard deviation (SD), which were acquired in at least three independent experiments. The statistical significances of differences were analyzed by using analysis of variance or Student's *t*-test.

## 3. Results

### 3.1. Overexpression of ROR*γ* in Liver Cancer Patients

**Figure 1:**
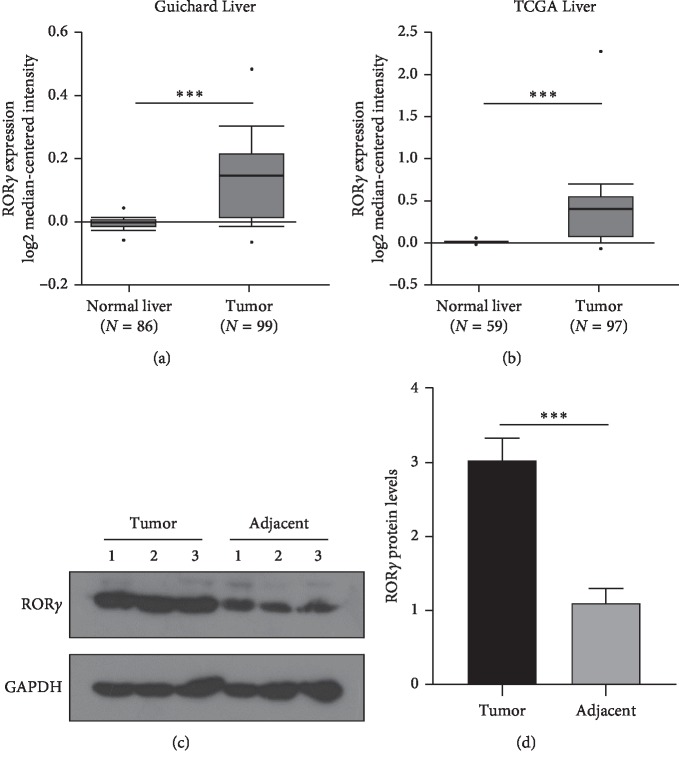
ROR*γ* was overexpressed in liver cancer. (a) The Guichard Liver was assessed from the Oncomine database, and the relative mRNA level was analyzed with the median-centered intensity method, and the adjacent liver tissues were considered as normal liver tissues and taken as the control group. (b) The TCGA database of liver cancer was subjected to analyzing the mRNA levels of ROR*γ*, and the adjacent liver tissues were considered as normal liver tissues and taken as the control group. (c) Three patients with liver cancer were subjected in this study, and the tumor tissues and corresponding adjacent normal liver tissues were applied to examine the protein levels of ROR*γ* with western blotting. (d) The relative quantitative protein intensity of ROR*γ* was evaluated with Quantity One software, and the data are expressed as mean ± standard deviation (SD), *n* = 3. ^*∗∗∗*^*P* < 0.001 was considered statistically significant.

A previous study revealed that ROR*γ* has a different regulation mechanism in different cancers. The study of ROR*γ* in liver cancer has not been reported yet. However, ROR*γ*t as a truncated variant was reported overexpressed in peripheral blood mononuclear cells of HCC patients. Then, we examined the ROR*γ* expression of liver cancer. As seen in Figure 1(a), compared with 86 cases of normal liver tissues, ROR*γ* mRNA levels were significantly increased in the 99 cases of liver tumor tissues. And a similar result was confirmed in the TCGA liver database (Figure 1(b)). To further confirm the overexpression of ROR*γ* in the liver tumor tissues, 3 pairs of patients with liver cancer in tumor tissues and corresponding adjacent normal tissues were subjected to western blotting; the protein expression of ROR*γ* was also enhanced remarkably (Figures 1(c)–1(d)). These results revealed that ROR*γ* was significantly increased in liver tumor at both protein and mRNA levels, suggesting that ROR*γ* may play a potential role in the occurrence and development of liver cancer.

### 3.2. Downregulation of ROR*γ* Promoter Methylation Activity in Liver Tumor

**Figure 2:**
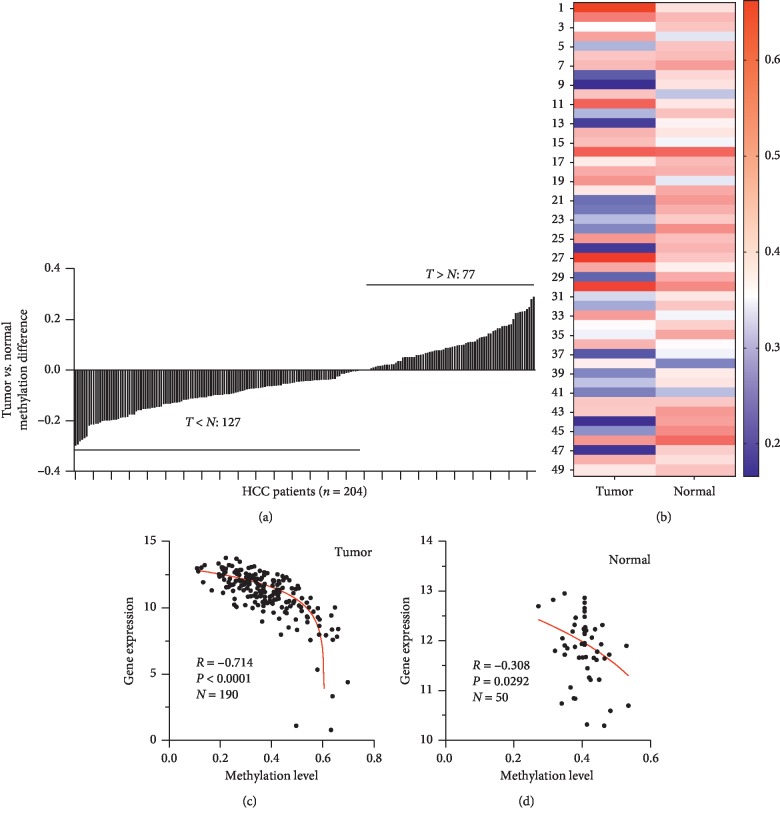
The promoter methylation levels of ROR*γ* were downregulated in liver tumors. (a) A total of 204 liver cancer patients were examined the ratio of promoter methylation levels of ROR*γ* in liver (T) tumor tissues and (N) normal liver tissues. (b) A total of 49 patients with methylation level data in tumor and corresponding normal tissues were subjected to analysis, and the result is represented as a heatmap. (c, d) The correlation between promoter methylation levels and gene expression levels of ROR*γ* was evaluated in both (c) liver tumor tissues and (d) normal liver tissues.

In our bioinformatics and western blotting analysis of ROR*γ* in liver cancer, we confirmed that ROR*γ* was overexpressed in the liver tumor tissues. Next, we tried to explore the overexpression of ROR*γ* in liver cancer, as methylation regulation was reported as an important regulatory mechanism for gene expression. Then, we hypothesized that methylation regulation may play an important role in the overexpression of ROR*γ* in liver tumor tissues. The TCGA analysis of the ROR*γ* promoter methylation activity of liver tumor tissues and normal liver tissues revealed that 62% of liver cancer patients express hypomethylation (Figure 2(a)). To accurately evaluate the promoter methylation levels of ROR*γ* in tumor and normal liver tissues, we selected 49 cases of patients with complete methylation levels both in tumor and corresponding normal tissues. The result showed that the promoter methylation levels of ROR*γ* were notably decreased in the tumor tissues (Figure 2(b)). To directly evaluate the association between methylation levels and gene expression levels of ROR*γ* in liver cancer patients, as the potential correlation between the gene expression and methylation level, we analyzed the correlation of ROR*γ* promoter methylation levels and its mRNA expression of 190 cases of liver tumor tissues and 50 cases adjacent normal liver tissues. The results showed that both in tumor and normal liver tissues, the promoter methylation levels of ROR*γ* were negatively correlated with its mRNA expression (Figures 2(c)–2(d)). Taking this, we believed that the promoter methylation regulation might be an important reason for the overexpression of ROR*γ* in liver tumor tissues.

### 3.3. Liver Cancer Patients with HBV Infection Representing Higher ROR*γ* Expression

**Figure 3:**
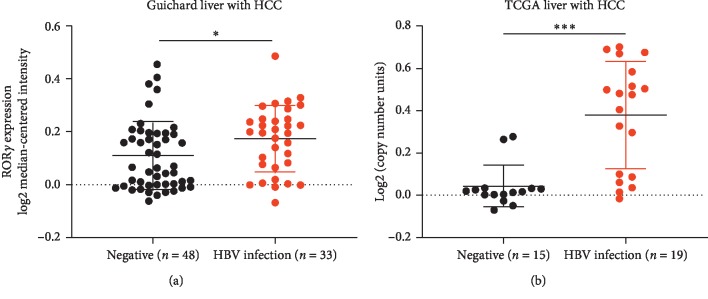
ROR*γ* expression was enhanced in liver tumor tissues with HBV infection. (a) The Guichard Liver of Oncomine database was divided into two groups, 48 patients were confirmed to have no HBV infection and 33 patients were confirmed to have HBV infection. (b) 34 patients from TCGA liver cancer database were included to evaluate the ROR*γ* levels, wherein 19 patients with HBV infection and 15 patients represented negative HBV infection. The data are expressed as mean ± standard deviation (SD). ^*∗*^*P* < 0.05, ^*∗∗∗*^*P* < 0.001 were considered statistically significant.

ROR*γ* was overexpressed in liver tumor. In order to deeply study the expression of ROR*γ* in different subtypes of liver cancers, as hepatitis virus infection is a major risk factor of HCC, especially the hepatitis B virus (HBV) infection widely occurs in Asia, we examined the ROR*γ* expression in hepatitis virus infection or negative infection in liver cancer patients. Interestingly, we found the expression of ROR*γ* was higher in these liver cancer patients with HBV infection than in those without hepatitis virus infection (Figure 3(a)). And, a similar phenomenon was revealed in the analysis of the TCGA database (Figure 3(b)). These results suggested that hepatitis virus infection might mediate the ROR*γ* expression through regulation of its coactivators in liver cancer.

### 3.4. HBx Increased the Expression of ROR*γ*

**Figure 4:**
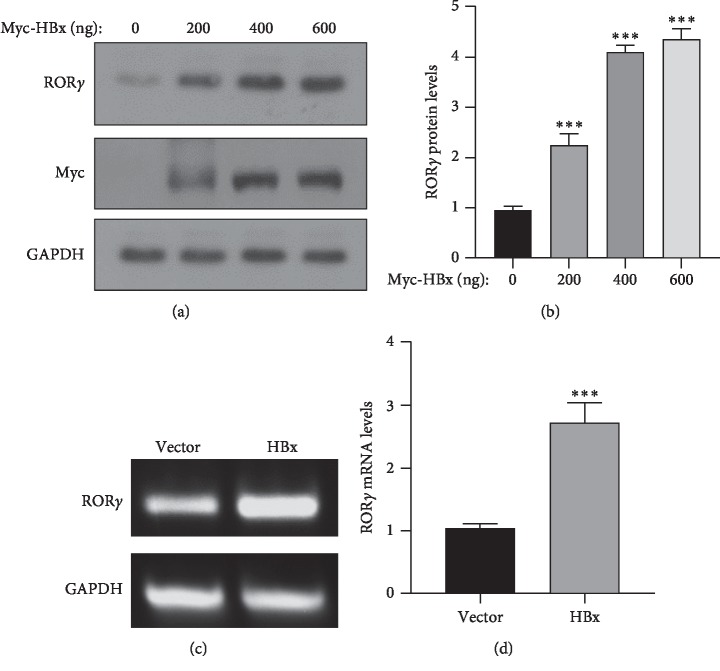
HBx can upregulate the expression of ROR*γ*. (a, b) The HepG2 cells were transfected with Myc-HBx plasmid in increased concentration, and western blotting was subjected to analyze the protein expression of ROR*γ*. (c, d) HepG2 cells were transfected with HBx-overexpressing and vector plasmids, and the mRNA levels of ROR*γ* were examined with RT-PCR. The Quantity One software was used for quantitative analysis. The data are expressed as mean ± standard deviation (SD), *n* = 3. ^*∗∗∗*^*P* < 0.001 was considered statistically significant.

Considering the higher expression of ROR*γ* in those liver cancer patients with HBV infection, HBV may be involved in the regulation of ROR*γ*. And hepatitis B virus X (HBx) protein is the most important factor of HBV-mediated liver cancer progression. Thus, we hypothesized that HBx may play an important role in the regulation of ROR*γ*. To confirm the hypothesis, HBx-overexpressing plasmid was transfected into the HepG2 cells and examined the expression of ROR*γ*. As the results showed, HBx can increase the protein levels of ROR*γ* in a dose-dependent manner (Figures 4(a) and 4(b)). Next, we also examined the transcriptional activity of ROR*γ* with or without HBx. The RT-PCR result revealed that HBx can enhance the mRNA level of ROR*γ* (Figures 4(c) and 4(d)). These results suggest that HBx may increase the ROR*γ* expression through the promotion of the transcriptional activity.

### 3.5. Promoter Activity of ROR*γ* Was Activated by HBx

**Figure 5:**
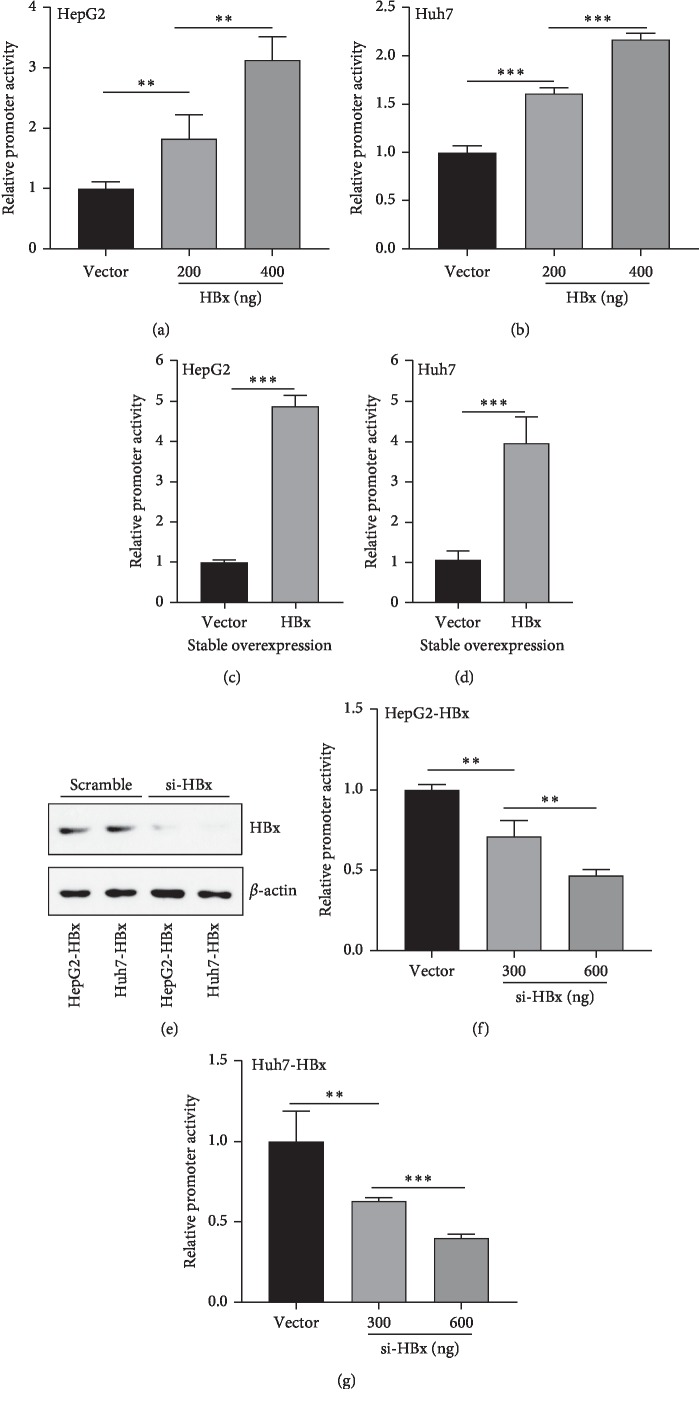
HBx can increase the promoter activity of ROR*γ*. (a) HepG2 and (b) Huh7 cells were transfected with pGL3-ROR*γ*, complemented with HBx plasmid. After 24 h transfection, the cells were subjected to analyze the reporter activity with the luciferase reporter system. (c) The stale overexpressing HBx cells and the vector controls of HepG2 and (d) Huh7 cells were transfected with pGL3-ROR*γ* reporter plasmid and Rellina plasmid, and the luciferase activity was detected by the dual-luciferase reporter system. (e) The knockdown efficiency of HBx was assessed in the HBx-stable overexpressing cells of HepG2 and Huh7. (f) The stable HBx-overexpressing HepG2 and (g) Huh.7 cells were transfected with pGL3-ROR*γ* reporter plasmid and cotransfected with siRNA against HBx. With 24 h culture, the promoter activity of ROR*γ* was detected by the luciferase reporter value. The data are expressed as mean ± standard deviation (SD), *n* = 4. ^*∗∗*^*P* < 0.01, ^*∗∗∗*^*P* < 0.001 were considered statistically significant.

As described earlier in this study, HBx can increase the expression of ROR*γ* in protein and mRNA levels, suggesting that HBx could regulate ROR*γ* through the transcriptional regulation mechanism. To confirm the hypothesis, we conducted the luciferase reporter of ROR*γ* promoter. As Figures 5(a) and B showed, HBx could increase the promoter activity of ROR*γ* in HepG2 and Huh7 cells in a dose-dependent way. Similarly, the promoter activity of ROR*γ* was also enhanced in the stable HBx-overexpressing cells (Figures 5(c) and 5(d)). To further confirm the regulation of HBx on the promoter activity of ROR*γ*. The HepG2-HBx and Huh7-HBx cells, which were stably overexpressing HBx, were subjected to HBx siRNA, and the efficiency of knockdown was confirmed at the protein level by western blotting (Figure 5(e)). Furthermore, we observed the luciferase activity of ROR*γ* promoter was significantly decreased in a dose-dependent manner (Figures 5(f) and 5(g)). Thus, HBx could activate the promoter activity of ROR*γ*. Taken these results, ROR*γ* was overexpressed in liver tumors, and HBx could increase ROR*γ* expression, we believed that HBx can increase ROR*γ* expression through activation of promoter activity.

### 3.6. ROR*γ* Promotes the Migration of Liver Cancer Cells

**Figure 6:**
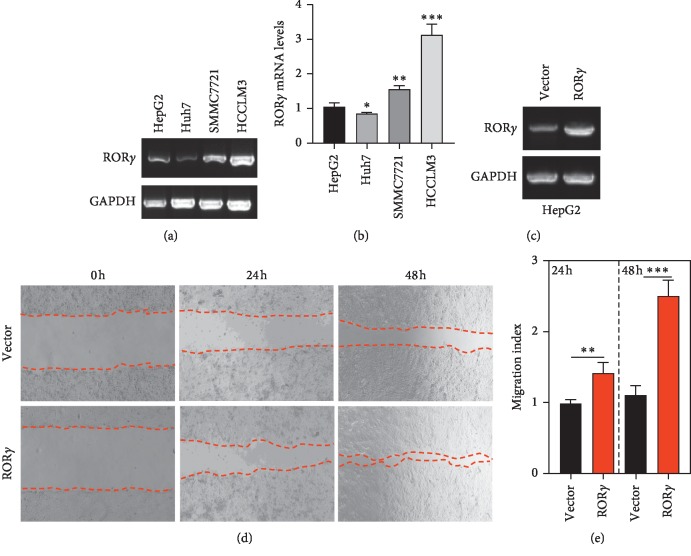
ROR*γ* promotes the migration activity of HepG2 cells. (a) The expression of HepG2, Huh7, SMMC7721, and HCCLM3 cell lines was evaluated with RT-PCR. (b) The relative quantitative mRNA levels of ROR*γ* were analyzed with Quantity One. (c) HepG2 cells were transfected with lentiviral particles of ROR*γ* and control vector, and with 24 h culture, the cells were subjected to RT-PCR to confirm the efficiency of overexpression of ROR*γ*. (d) HepG2 cells were transfected with lentiviral particles, and further cultured for indicated times, the convergence of cells were recorded. (e) The migration index was calculated with the ratio of the scratch area between ROR*γ* overexpression and vector cells by Image J software. The data are expressed as mean ± standard deviation (SD), *n* = 3. ^*∗*^*P* < 0.05, ^*∗∗*^*P* < 0.01, ^*∗∗∗*^*P* < 0.001 were considered statistically significant.

ROR*γ* was overexpressed in liver tumor tissues, and the ROR*γ* expression was mediated by the HBx, an important oncogene in the development of liver cancer; these data suggest that ROR*γ* may act as a cancer promoter in liver cancer. To further validate the function of ROR*γ*, ROR*γ* was detected in HepG2, Huh7, SMMC7721, and HCCLM3 cells. And HCCLM3 represented the highest expression of ROR*γ* (Figures 6(a) and 6(b)), and as the previous report, among the four cell lines, HCCLM3 was the most malignant grade with higher migration activity [25]. Thus, whether ROR*γ* has a potential role in the migration process of liver cancer cells is unclear. Then, HepG2 cells were selected to the next study. The lentiviral particles of overexpressing ROR*γ* and control vector were confirmed with RT-PCR (Figure 6(c)), and the effect of ROR*γ* on the migration activity of liver cancer cells was evaluated by wound-healing assay. The results showed that overexpression of ROR*γ* could significantly promote the migration of HepG2 cells after incubation with lentiviral particles for 24 h. And for another 24 h culture, the migration index was further enhanced (Figures 6(d) and 6(e)).

### 3.7. ROR*γ* Enhances the Proliferation Activity of Liver Cancer Cells

**Figure 7:**
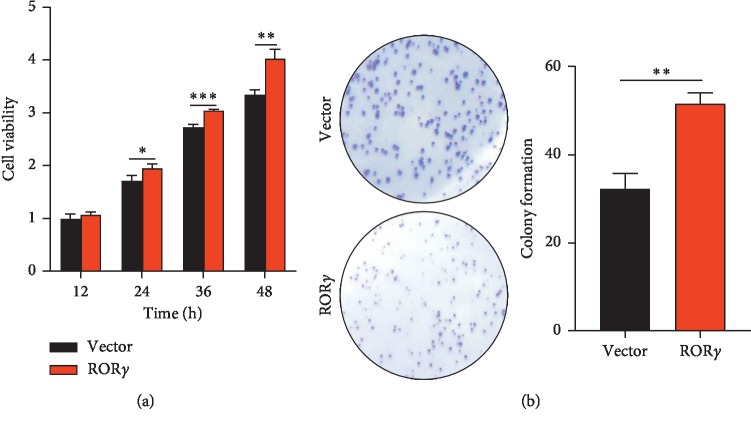
ROR*γ* accelerates the proliferation of HepG2 cells. (a) HepG2 cells were cultured in 96-well plates and the cells were treated with lentiviral particles of overexpressing ROR*γ* and control vector. With indicated treatment time, the cell viability was assessed by CCK-8 assay. (b) HepG2 cells were treated with lentiviral particles overexpressing ROR*γ* and control vector for indicated times, and then the colony number was calculated with Image J after crystal violet staining. The data are expressed as mean ± standard deviation (SD), *n* = 3. ^*∗*^*P* < 0.05, ^*∗∗*^*P* < 0.01, ^*∗∗∗*^*P* < 0.001 were considered statistically significant.

ROR*γ* has a potential role in the migration activity of liver cancer cells, and the effect of ROR*γ* on the proliferation activity was examined. As Figure 7(a) revealed, cell viability showed no significant difference after treatment with ROR*γ*-overexpressing lentiviral particles for 12 h. After treatment with the above for 24 h, cell viability was enhanced by overexpression of ROR*γ* (Figure 7(a)). To confirm the effect of ROR*γ* on the regulation of proliferation in liver cancer cells, the colony formation assay was used in this study. The results showed the colony number was increased after overexpression of ROR*γ*. Thus, the cell viability and colony formation assay both represented that ROR*γ* accelerated the proliferation activity of liver cancer cells.

## 4. Discussion

The current study of ROR*γ* in cancers remains still unclear. ROR*γ* was reported as an oncogene in non-small-cell lung cancer and melanoma cancer [13, 15]. However, ROR*γ* was also described as a tumor suppressor gene in breast cancer, suggesting that ROR*γ* performs a different function in different organs [11, 26]. But the recent study also revealed that even in the same type of cancer, ROR*γ* was reported to perform a different function. Considering ROR*γ*t as an important regulator in the immune regulation network, the immune system was a crucial aspect of the tumor microenvironment. Similarly, ROR*γ* is different from ROR*γ*t at the N-terminal, and ROR*γ* is also selectively expressed in the Th17 cells and involved in the control of Th17 differentiation [27]. These contribute to the different functions in cancers. Up to date, about the function of ROR*γ* in liver cancer was not reported yet. In this study, we intended to study the expression of ROR*γ* and its significance in liver cancer cells. The previous study showed that ROR*γ*t was highly expressed in peripheral blood lymphocyte of liver cancer patients [18], ROR*γ* as a similar isoform, the expression and its significance have not been reported, and then we explored the expression of ROR*γ* in liver cancer. The bioinformatics analysis and western blotting showed that ROR*γ* was highly expressed in the liver tumor tissues compared with the adjacent normal liver tissues (Figure 1). More interestingly, we firstly found that the methylation levels of ROR*γ* in liver tumor tissues were downregulated compared with the normal liver tissues (Figures 2(a) and 2(b)). Similar to the current study, the regulation of methylation in the gene expression was an important method [28]. Also, the correlation analysis confirmed that the overexpression of ROR*γ* in the liver tissues was due to its hypomethylation levels of the promoter (Figures 2(c) and 2(d)), as numerous studies have revealed that the aberrant DNA methylation was deeply involved in the occurrence and development of some cancers [1, 29, 30]. In this study, we showed that the aberrant expression of ROR*γ* in liver cancer tissues and the methylation regulation contributed to the overexpression of ROR*γ* in liver tumor tissues.

As hepatitis B virus (HBV) infection is a major risk factor of liver cancer [31], hepatitis B virus X protein (HBx) is a major protein for HBV-related liver cancer [32]. And the former study revealed that ROR*γ* may be involved in the development of liver disease [33]. Then, to evaluate the correlation between HBx and ROR*γ*, we test the correlation between both the molecules. The results showed ROR*γ* expression was further increased in the HBV-positive liver cancer tissues (Figure 3). And overexpression of HBx in liver cancer cells, the expression of ROR*γ* was significantly increased in both protein and mRNA levels (Figure 4). The transcriptional regulation is an essential manner for gene levels. Then, we hypothesized that HBx could promote the transcriptional activity of ROR*γ*. And the results also confirmed our hypothesis. Furthermore, to confirm the results, we also knockdown HBx in the HBx-stable expressing cells (Figure 5). And these results demonstrated that ROR*γ* was a novel regulator in HBV-positive liver cancer. However, given that HBx was a coactivator of transcription regulation, HBx could activate some signaling pathways, so it is an important direction to study the regulation of HBx on ROR*γ* in further studies. Another important question is that the methylation level of ROR*γ* promoter was downregulated in the liver tumor tissues, and the promoter methylation regulation might contribute to the overexpression of ROR*γ* in liver tumor tissues, and we also reported HBx mediated the ROR*γ* expression. Previous reports show that HBx can regulate some genes via DNA methylation, especially in the methylation of the promoter [34, 35]. Then, whether HBx could affect the promoter methylation of ROR*γ* needs further evaluation.

To confirm the significance of ROR*γ* in liver cancer, we evaluated the function of ROR*γ* in the regulation of migration and proliferation activity in liver cancer cells. The wound-healing assay revealed that overexpression of ROR*γ* could enhance the migration activity of liver cancer cells (Figure 6). Furthermore, the cell viability and colony assays indicated that ROR*γ* has positive regulation in the process of proliferation (Figure 7). These functional tests initially evidenced that ROR*γ* performed as an oncogene in the liver cancer cells.

In conclusion, this study demonstrated that ROR*γ* was highly expressed in the liver cancer tissues and the disorder of methylation is an important factor contributing to the overexpression of ROR*γ*. More interestingly, we firstly revealed that ROR*γ* was close with HBV-related liver cancer and HBx could increase the expression of ROR*γ* through the promotion of promoter activity. And *in vitro* studies also showed ROR*γ* was a positive regulator in the migration and proliferation of liver cancer cells, suggesting that ROR*γ* was a novel target for liver cancer therapy, especially for the HBV-positive liver cancer.

## Data Availability

Data are available from the corresponding author.
